# System Analysis of LWDH Related Genes Based on Text Mining in Biological Networks

**DOI:** 10.1155/2014/484926

**Published:** 2014-08-27

**Authors:** Mingzhi Liao, Yingbo Miao, Liangcai Zhang, Yang Wang, Rennan Feng, Lei Yang, Shihua Zhang, Yongshuai Jiang, Guiyou Liu

**Affiliations:** ^1^College of Life Sciences, Northwest A&F University, Yangling, Shaanxi 712100, China; ^2^College of Pharmacy, Nankai University, Tianjin 300071, China; ^3^College of Bioinformatics Science and Technology, Harbin Medical University, Harbin 150081, China; ^4^Department of Statistics, Rice University, 6100 Main Street, Houston, TX 77005, USA; ^5^Department of Nutrition and Food Hygiene, School of Public Health, Harbin Medical University, Harbin 150081, China; ^6^College of Life Science, Anhui Agricultural University, Hefei 230036, China; ^7^Genome Analysis Laboratory, Tianjin Institute of Industrial Biotechnology, Chinese Academy of Sciences, Tianjin 300308, China

## Abstract

Liuwei-dihuang (LWDH) is widely used in traditional Chinese medicine (TCM), but its molecular mechanism about gene interactions is unclear. LWDH genes were extracted from the existing literatures based on text mining technology. To simulate the complex molecular interactions that occur in the whole body, protein-protein interaction networks (PPINs) were constructed and the topological properties of LWDH genes were analyzed. LWDH genes have higher centrality properties and may play important roles in the complex biological network environment. It was also found that the distances within LWDH genes are smaller than expected, which means that the communication of LWDH genes during the biological process is rapid and effectual. At last, a comprehensive network of LWDH genes, including the related drugs and regulatory pathways at both the transcriptional and posttranscriptional levels, was constructed and analyzed. The biological network analysis strategy used in this study may be helpful for the understanding of molecular mechanism of TCM.

## 1. Introduction

Traditional Chinese medicine (TCM) is one important component of the medical drug system, which can treat disease systematically and is effective at recuperating the balance of the whole body in patients using herbal formulas (Fang-Ji in Mandarin) [1]. TCM usually uses several medicinal herbs that have different roles. The ingredients of these herbs have been organized into certain formulas that may have potential complex interaction effects. Considering the different natural properties of herbs, including hot, warm, cool, and cold, the interactions among herbs will be difficult to be understood [2, 3].

In order to understand the complex effects of TCM formulas in the whole body, research about molecular targets of formula may be necessary. Some previous studies have found important target genes of TCM. Li et al. analyzed the target genes of TCM [4–6]. Based on the target prediction of TCM compounds, Liang et al. tried to construct the network about one case of TCM, which represents the newest progress of network approach in pharmacology [7]. Some researches tried to construct the relationship between TCM and disease in silico [8, 9]. Fang et al. collected the target genes of TCM into a database TCMGeneDIT and analyzed those genes [10]. Furthermore, some researchers have generated a comprehensive database for TCM [11]. Based on the accumulation of TCM modernization, Chen group provided web servers for virtual screening and de novo drug design [12, 13]. However, suitable methods for determining how the target genes work in the complex environment of the whole body have not been developed.

Complex biological systems can be broken down into interacting networks composed of nucleotides, RNA, proteins, drugs, foods, and so forth. Studies of biological networks are increasing the understanding of the mechanism of biological systems [14, 15]. With the rapid development of computational systems biology, especially network biology, the mechanisms study about how TCM target genes work has changed from “one drug, one target” to “multidrugs, multitargets, biological networks” [16–18]. Based on these concepts, some studies constructed the drug targets network with PPINs [19]. Li group made their efforts to analyze the topological properties of drug targets in PPINs [20, 21]. Yang et al. also analyzed the topological characteristic of toxin targets in PPINs [22]. Though this research model is still in its infancy, computational systems pharmacology has also been highlighted in the medical drug development fields [23, 24]. Previous studies attempted to construct and analyze the networks about TCM [4, 25]. Considering the usefulness of analyzing biological networks to determine the complex effects of TCM target genes globally, we performed experiments to explore the topological properties of TCM in biological networks. These experiments had been previously applied to study social networks in social sciences [26].

In this study, candidate target genes of “Liuwei-dihuang” (LWDH, also known as Rehmannia Six, Six-Ingredient Rehmannia, or Rokumigan, a famous TCM formula that may nourish the balance of kidney yin yang) were obtained from previous studies using natural language mining technology. Then, the topological properties of LWDH genes were analyzed in PPINs. Interestingly, the LWDH genes were found to have high intensive centrality in PPINs. This indicates that the LWDH genes may have more important roles in the biological process than other genes. We also found that the distances within LWDH genes were smaller than other genes, which indicates that the LWDH genes may respond rapidly and synergistically to the common biological stimulation. For further understanding the inner molecular mechanism, a comprehensive network of LWDH genes was constructed and some modules were found.

## 2. Materials and Methods

### 2.1. Extraction of LWDH Genes Using Text Mining Technology

Considering the complexity of LWDH components and their complex interactions* in vivo*, little is known about the target genes of LWDH. The complex components of LWDH are comprised with six herbs. But their genomes of the six herbs that are components of LWDH have not been sequenced. Thus, determining the target genes of LWDH was critical for understanding the molecular mechanisms of LWDH. The existing research regarding LWDH genes is scattered in different groups around the world, even though the total number of LWDH target genes is small. Currently, researchers normally choose candidate genes for study by extensive literature review. However, scientific literature is now growing rapidly along with the development of the life science. The database of PubMed biomedical literature has over 23 million citations for biomedical literature from MEDLINE, life science journals, and online books. Thus, it is impossible for a researcher of a specific area to read all the literatures in his field, not to mention the papers in the related field. In this study, we obtained candidate LWDH target genes for study using the text mining technology.

We obtained LWDH genes for study by mining and analyzing the biomedical literature in PubMed with natural language processing technology. Considering the composition of LWDH, we extracted LWDH genes according to the components. As there are six herbs of LWDH, so we first decomposed LWDH with several search terms correspondingly, including Liu-wei-di-huang (LWDH, also known as Rehmannia Six, Six-Ingredient Rehmannia, or Rokumigan), Shan-zhu-yu (Fructus Corni), Ze-xie (Rhizoma Alismatis), Dan-pi (Cortex Moutan), Di-huang (Radix Rehmaniae), Fu-ling (Poria Cocos), and Shan-yao (Rhizoma Dioscoreae), and combined these search terms with “or” or “and” logic operators. Second, we extracted literature about Homo sapiens with the search terms in PubMed using eSearch and eFetch with the SQL statement. Third, we analyzed the literature with MEDLINE format files to get the gene name using the gene/protein names recognition software AbGene and filed the results manually [27]. At last, all the data were stored in the MySQL 5.0.90 database and reorganized into one list.

### 2.2. Protein-Protein Interaction Network Construction

The PPINs were constructed with a large number of proteins and their interactions, where the nodes denote proteins and the edges denote interactions between different nodes. PPINs are the most frequently used biological networks for research in computational system biology. PPINs are undirected networks that can be detected using multiple methods, including high-throughput and low-throughput experiments, such as yeast two-hybrid, affinity capture-MS, synthetic lethality, and reconstituted complex. In order to illustrate the robustness of our results to the primary dataset, we generated two different original datasets. The first is BioGrid and the other is HPRD [28, 29]. Considering the false position rate, the high-throughput data for both datasets were filed out. In this study, the self-interactions were also eliminated.

### 2.3. The Topological Analysis of PPINs

To explore the roles that LWDH genes play in PPINs, this study focused on the centrality analysis of nodes. AS LWDH has the therapeutic effects of recovering the balance of kidney yin and yang in the complex whole-body environment, we hypothesized that the LWDH genes products in PPINs have different roles from other normal proteins. The centrality of one node in network has different measurements, including degree, betweenness, and clustering coefficient (Table 1) [30, 31]. We obtained as many centrality measurements as possible to explore the properties of LWDH genes. We obtained a total of 9 centrality measurements. Among the 9 measurements, degree is the basic element in the topological analysis which was used to detect the number of edge links to the node. Besides, all other topological properties can be used to measure the centrality of nodes in biological networks. In general, the higher the centrality of one node, the more important roles it plays in biological networks. For detailed description, we took some properties as examples to illustrate their meanings. Average shortest path length (ASP) is defined as the average length between a node and all the nodes in biological networks. Closeness centrality is defined as the reciprocal of the average shortest path length of one node which can be used as a measure of how fast information spreads from a given node to all other reachable nodes in biological networks. In undirected biological networks (such as PPINs), clustering coefficient of a node is defined as the proportion of the observed connections between the neighbors of this node against the maximum number of possible connections among them. Clustering coefficient is used to indicate the close extent of the local neighborhood of one node. Topological coefficient is a relative measurement of the tendency of one node in biological networks to have shared interactive partners with other nodes. For more in-depth interpretation of these concepts, one can get the exact definitions of these topological properties from Table 1.

In this study, we also paid close attention to the contacts within LWDH genes. There is one hypothesis that if the LWDH genes studies here had the same functions and acted on the same biological processes, it is expected that the distances among LWDH genes should be shorter than the average distance in background network. A shorter distance between two nodes in network will be helpful for information communication. Thus, this study will analyze the shortest path length among the LWDH genes.

Both the centrality analysis and shortest path length comparison were performed using the software Cytoscape version 3.0.0 [32, 33].

### 2.4. Construction and Analysis of Comprehensive Network about LWDH Genes

**Figure 1:**
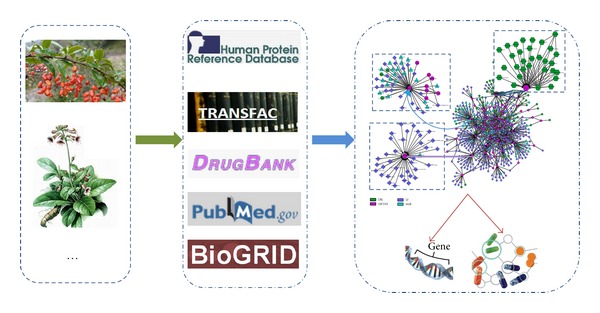
Workflow diagram of our approach to construct comprehensive biological network about LWDH genes. The left frame represents the component of LWDH. After the mining of related genes of LWDH based on natural language technology in PubMed, comprehensive information was obtained, including protein-protein interaction, transcription regulation, drug-drug interaction, and drug-target interaction (middle frame). At last, the comprehensive network of LWDH genes was constructed (right frame).

An integrated biological network was constructed (Figure 1). Firstly, we got the drug targets and drug-drug interaction information from DrugBank [34]. Second, the transcriptional regulation information was obtained from Transfac [35]. Third, we also detected the effects of posttranscriptional regulation on LWDH genes with microRNAs (miRNAs) and the related targets of miRNAs were from Tarbase, which only collected the results of experimental verification [36].

## 3. Results

### 3.1. LWDH Genes

Based on the text mining in PubMed, we first got all the references about LWDH. There are 151 816 entries, including 3228 references, 107 species, and 145 099 genes; see Table S1 in Supplementary Material available online at http://dx.doi.org/10.1155/2014/484926. As we focused on the genes that play roles in human, we considered genes on other species as false positive results. After the filter of species, we got total of 107 manually collected entries from which we can get LWDH genes with different searching conditions, including 96 unique genes related to LWDH in 61 literatures (Table S2). From the results, we found that di-huang is the core component of LWDH and had been most thoroughly investigated. Di-huang related genes were studied in 101 times. These studies included 93 unique genes in 56 literatures. On the other hand, no research was found for ze-xie related genes. When the LWDH genes were mapped to the most component in PPINs, the total numbers of LWDH genes remain 71 and 65 in BioGrid and HPRD, respectively.

To verify the function of the LWDH genes, we performed a function enrichment analysis using gene ontology (Table 2). From the results, we found that the regulation of cell apoptosis and death is the main function of LWDH genes, which is consistent with previous study [37].

### 3.2. Protein-Protein Interaction Networks

**Figure 2:**
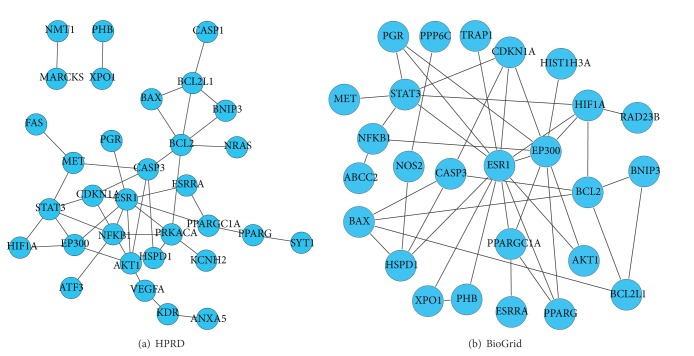
Subnetwork of LWDH genes from BioGrid and HPRD.

There were a total of 10,996 nodes and 54,433 edges in BioGrid primary. After removing the isolated nodes, there were 10,768 nodes and 52,993 edges. Similarly, there were a total of 9,453 nodes and 36,888 edges in HPRD primary, and after removing the isolated nodes, there were 9,205 nodes and 36,748 edges. The LWDH genes that were mapped to the PPINs formed subnetworks that showed the core genes and their information communication path between them.  Figure 2 shows that* ESR1*,* STAT3*,* EP300*, and so forthhave important positions in two subnetworks from both the BioGrid and HPRD datasets, respectively. The results indicate that these genes have important functions in the LWDH interaction environment.

### 3.3. Centrality of LWDH Genes in PPINs

In the centrality analysis of PPINs, 9 measurements in Table 1 were compared between the nodes which belong to LWDH genes (abbreviation as LWDH) and the nodes which do not belong to LWDH genes (abbreviation as Other). All the computations were processed with *t*-test in *R* program. Results show that the LWDH genes have higher centrality than the other nodes in the PPINs (Tables 3 and 4). The BioGrid results showed that 8 out of the 9 measurements of the LWDH genes were significantly greater than the other nodes (Table 3). The HPRD dataset also supports the same tendency (Table 4).

### 3.4. Shortest Path Length Comparison

**Figure 3:**
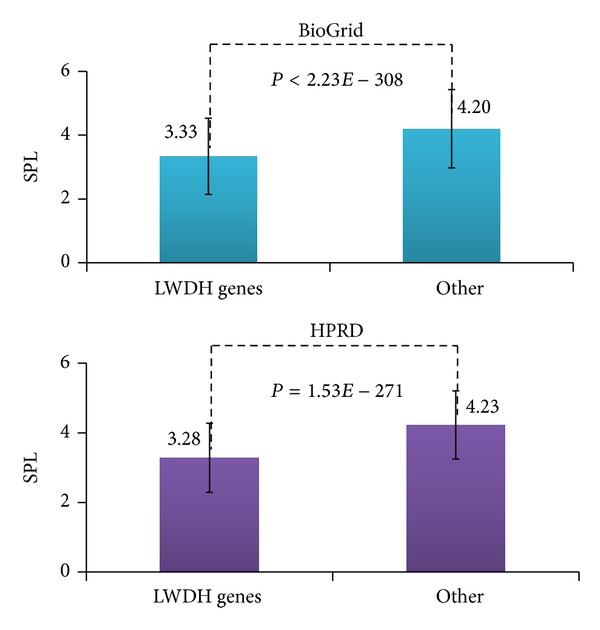
Comparison of the shortest path length between pairs of LWDH genes and pairs of other nodes. The left groups represent pairs of genes that are related to LWDH, and the right groups represent pairs of genes that are not related to LWDH. The measurement of SPL means the shortest path length of gene pairs in each group.

In order to explore the modularity within LWDH genes, we processed the computation about the shortest path length. As one famous herbal formula, LWDH is widely used and has been proved to be useful. If the LWDH gene is detected correctly, it is expected that the LWDH genes may show modularity and have closer connections inside themselves than the random chosen ones. In order to verify this hypothesis, the connection extent between each other among LWDH genes was compared with the background PPINs used shortest path length. As the expectation, Figure 3 supports that hypothesis. In the BioGrid datasets, the mean distance between the LWDH genes was 3.33, and if two nodes in the PPINs were chosen randomly, the expected distance was 4.20 (with *P* < 2.23*E* − 308) (upper panel of Figure 3). Similar to the BioGrid results, the mean shortest path length of the LWDH genes in the HPRD was 3.28, compared to 4.23 in the random situation (with *P* = 1.53*E* − 271) (lower panel of Figure 3).

### 3.5. Comprehensive Network of LWDH Genes

**Figure 4:**
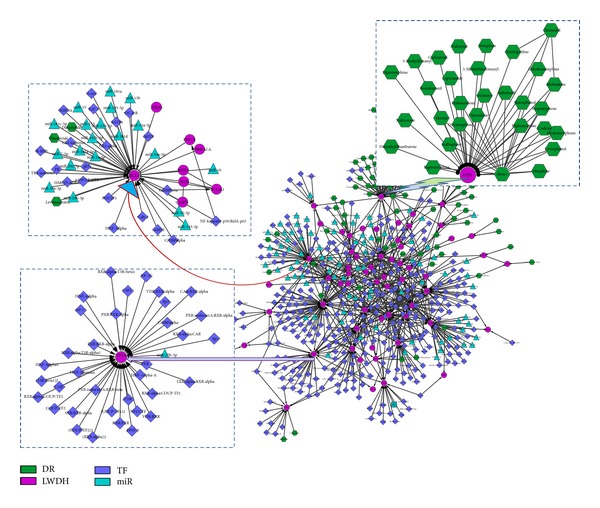
Comprehensive network of LWDH genes. Green represents drugs; purple represents LWDH genes; blue represents TFs; cyan represents miRNAs.

The network reflects the impacts of both biological molecules and synthetic compounds (Figure 4). It contains 4 types of nodes, including 62 LWDH genes, 301 transcriptional factors (TFs), 85 miRNAs, and 83 chemical compound drugs. Among these nodes, there were 5 types of edges, including 63 protein-protein interactions (from both HPRD and BioGrid), 516 TF transcriptional regulations, 113 miRNA posttranscriptional regulations, 106 drug-target relationships, and 40 drug-drug interactions (Table S3). From the network, we obtained a panoramic view of the molecular interaction pathway of LWDH genes. Furthermore, the modules around the LWDH genes were found in the comprehensive networks. According to the types of nodes around the LWDH genes, the modules were classified into three types: the first one is drugs (drug-module); the second one is TFs (TF-module); the third one is a mix of TFs and miRNAs (mixed-module). As a typical example of drug-module,* ATF3* was targeted by a lot of drugs (upper right of Figure 4). Because* ATF3 *relates with both LWDH and lots of drugs, there should be common biological pathway through* ATF3* that is shared by LWDH and these drugs. On the basis of common pathway about* ATF3*, this research may provide some novel molecular mechanism about the treating of disease with LWDH.* CYP3A4* is one typical TF-module, which is targeted by lots of TFs (lower left of Figure 4). This result indicates that* CYP3A4 *may be a key crosstalk link to the pathways of these TFs* in vivo*. Considering the relationship between LWDH and these TFs through* CYP3A4*, we infer that LWDH implements its function of treating disease synergistically with these pathways of the TFs. As a TFs and miRNAs mixed-module,* BCL2* is around with lots of TFs and miRNAs (upper left of Figure 4). This means that* BCL2 *may play roles from both transcriptional and posttranscriptional regulation levels.

## 4. Discussion

Facing the difficulty of studying the molecular mechanism about TCM targets, this research provided some novel approaches to reveal new characters of TCM genes in biological network. Through the example of LWDH, we got their candidate target genes based on natural language text mining technology and found that they may play important roles in PPINs. We also found that LWDH genes have relatively close communication in common biological process. Understanding the characteristics of TCM herbal formulas in complex biological networks, particularly through the molecular mechanism exploration, will not only benefit the modernization of TCM but may also be helpful for the development of new drugs [2, 17].

To address the lack of known target genes of LWDH, this study extracted all the known genes related to LWDH in Homo sapiens using natural program technology and deemed them LWDH genes. We used OMIM datasets as the criterion for validating the effectiveness of text mining methods for obtaining LWDH genes [38] (Table 5). The results showed that LWDH genes were mainly related to diseases of nosohemia and cancer, which is consistent with previous research [7, 39, 40]. Compared with Liang et al. research, our work provided all the genes in existing literatures and characterized the topological properties in biological networks firstly [7]. This strategy will be helpful for the traditional biological medicine research fields, which are still lacking genome information for the herbs that comprise LWDH. Using computational methods, this study obtained the LWDH genes from a large number of literatures rapidly. Considering the automation during the search of the literatures and the false positive results that were hidden in the literatures, the results of this study may need to be adjusted with new text searching technology and more abundant information in future.

Based on the extracted LWDH genes obtained from magnanimous literatures, we constructed two protein-protein interaction networks from different datasets and processed some topological analysis. Both analyses showed that the LWDH genes have higher centrality in PPINs, which indicate that they may play more important roles in complex whole-body environments. When one node has higher centrality properties in biological network, there would be more information flow through it. These nodes may be regarded as key components in network, which help to maintain the stabilization of the molecular pathway. This result is consistent with the concepts of TCM function “balance” in regard to yin and yang, because only the proteins in the center of PPINs may be convenient to get the global information of body and can quickly respond to the whole-body environment [2, 41].

Interestingly, we also found that there is a smaller distance between the LWDH genes than would occur randomly. This indicates that the function of LWDH is focused on some particular biological process and that LWDH target genes have sufficient and quick information interflow. This is consistent with the well-known function of LWDH to help maintain and restore the balance of kidney yin yang [41]. These results also indicate that the LWDH herbal components are grouped together as the small distances between the nodes in the PPINs which mean the concentration of function. If one TCM herbal formula is expected to have good effects in treatment, its components may all focus on one main function and execute a common biological process synergistically.

Based on system information, a comprehensive network of LWDH genes was constructed. From that network, the effects of both biological molecules and artificial chemical drugs were clearly detected. Besides, some interesting results were found. Three types of modules around the LWDH genes were extracted from the comprehensive network. Genes in drug-modules are candidate targets of LWDH in treating disease. Observing genes in TF-modules can help researchers to detect the molecular mechanism of LWDH* in vivo*. Genes in mixed-modules may be helpful for exploring the epigenetic effects of LWDH in posttranscriptional regulation.

## 5. Conclusions

This research describes a new approach to explore the molecular mechanisms of TCM in complex biological networks. The topological properties observed may be helpful for the characterization and prediction of TCM target genes. The shortest path length comparison may also provide some criteria to estimate the rationality of TCM herbal formulas. Different modules in the comprehensive network of LWDH genes may provide a global perspective regarding TCM molecular mechanisms. With the development of computational system biology, their advantages in processing big data of biological medicine will be more significant.

## Supplementary Material

These supplementary informations include the primary reference results of text mining, LWDH related genes in human and the comprehensive network of LWDH.

## Figures and Tables

**Table 1:** Topological definition used in the study.

Name	Definition	Description
ASP	$\frac{\sum_{j\in N}d_{ij}}{\left|N||N-1\right|}$	*N* denotes the set of all nodes in the PPI network; *d*_*ij*_ denotes the shortest path between node *i* and node *j*

Betweenness	$B_{i}=\overset{}{\underset{k\neq j\neq i\in V}{\sum }}\frac{\left|\sigma_{kij}\right|}{\left|\sigma_{kj}\right|}$	*B*_*i*_ represents the betweenness of node *I*, σ_*kj*_ denotes shortest paths between node pairs *k* and *j*, and σ_*kij*_ denotes that pass through the node *i*

Closeness	$\frac{1}{\text{avg}(L(n,m))}$	*L*(*n*, *m*) is the length of the shortest path between two nodes *n* and *m*. The closeness centrality of each node is a number between 0 and 1

Clustering coefficient	$\frac{2n_{i}}{\left(k_{i}(k_{i}-1)\right)}$	*n*_*i*_ is the number of links between all neighbors of node *i*

Degree	*k*_*i*_	The number of links to node *i*

Eccentricity	*E*	The maximum node eccentricity (*E*) can be described as the network diameter, which is the largest distance between two nodes

Radiality	*R* = *D* − ASPL + 1	This attribute is a node centrality index computed by the diameter (*D*) of a node *n*'s the connected component plus 1 and subtracting the average shortest path length (ASPL)

Stress	*p*_*i*_	*p*_*i*_ is the number of shortest paths passing through *i*

Topological coefficient	$t_{i}=\frac{\text{avg}(J(i,j))}{d_{i}}$	*t*_*i*_ represents the topological coefficient of node *I*; *J*(*i*, *j*) is the number of neighbors shared between the nodes *i* and *j*, plus one if there is a direct link between *i* and *j*. avg(*J*(*i*, *j*)) is the average value of *J*(*i*, *j*). *d*_*i*_ is degree of node *i*

**Table 2:** Functional enrichment analysis of LWDH genes in the biological process branch of GO.

GOID	Term	Count	Count%	*P* value	FDR
GO:0043066	Negative regulation of apoptosis	18	18.9	4.00*E* − 12	6.80*E* − 09
GO:0043069	Negative regulation of programmed cell death	18	18.9	5.00*E* − 12	8.50*E* − 09
GO:0060548	Negative regulation of cell death	18	18.9	5.30*E* − 12	8.90*E* − 09
GO:0042981	Regulation of apoptosis	23	24.2	1.10*E* − 10	1.90*E* − 07
GO:0043067	Regulation of programmed cell death	23	24.2	1.40*E* − 10	2.30*E* − 07
GO:0010941	Regulation of cell death	23	24.2	1.50*E* − 10	2.50*E* − 07
GO:0010033	Response to organic substance	20	21.1	5.30*E* − 09	9.00*E* − 06
GO:0006916	Antiapoptosis	12	12.6	1.20*E* − 08	2.10*E* − 05
GO:0042127	Regulation of cell proliferation	20	21.1	2.20*E* − 08	3.70*E* − 05
GO:0009719	Response to endogenous stimulus	15	15.8	2.70*E* − 08	4.70*E* − 05
GO:0070482	Response to oxygen levels	10	10.5	6.20*E* − 08	1.10*E* − 04
GO:0009725	Response to hormone stimulus	14	14.7	6.90*E* − 08	1.20*E* − 04
GO:0043065	Positive regulation of apoptosis	14	14.7	4.30*E* − 07	7.30*E* − 04
GO:0043068	Positive regulation of programmed cell death	14	14.7	4.60*E* − 07	7.90*E* − 04
GO:0010942	Positive regulation of cell death	14	14.7	4.90*E* − 07	8.30*E* − 04
GO:0001666	Response to hypoxia	9	9.5	5.90*E* − 07	1.00*E* − 03
GO:0048545	Response to steroid hormone stimulus	10	10.5	8.60*E* − 07	1.50*E* − 03
GO:0001776	Leukocyte homeostasis	6	6.3	1.30*E* − 06	2.20*E* − 03
GO:0042592	Homeostatic process	17	17.9	1.90*E* − 06	3.20*E* − 03
GO:0006915	Apoptosis	15	15.8	3.30*E* − 06	5.70*E* − 03
GO:0051384	Response to glucocorticoid stimulus	7	7.4	3.80*E* − 06	6.40*E* − 03
GO:0012501	Programmed cell death	15	15.8	4.00*E* − 06	6.70*E* − 03
GO:0043029	T cell homeostasis	5	5.3	4.40*E* − 06	7.40*E* − 03
GO:0045884	Regulation of survival gene product expression	5	5.3	4.40*E* − 06	7.40*E* − 03
GO:0008219	Cell death	16	16.8	5.40*E* − 06	9.10*E* − 03
GO:0016265	Death	16	16.8	5.80*E* − 06	9.90*E* − 03

**Table 3:** Centrality analysis of LWDH genes in BioGrid.

Measurements	LWDH	Other	*P* value	−log⁡10(*P* value)
ASP	3.322458	3.756768	7.13*E* − 12	11.14671
Betweenness	0.002336	0.000242	1.49*E* − 11	10.82709
Closeness	0.308365	0.27118	7.01*E* − 18	17.15445
Clustering coefficient	0.113448	0.156074	0.155779	0.807491
Degree	46.88732	9.596803	1.64*E* − 31	30.78599
Eccentricity	7.309859	7.731794	2.9*E* − 08	7.537242
Radiality	0.788867	0.749385	7.13*E* − 12	11.14671
Stress	5075568	500694.1	1.36*E* − 16	15.86601
Topological coefficient	0.129467	0.178003	0.032223	1.491832

**Table 4:** Centrality analysis of LWDH genes in HPRD.

Measurements	LWDH	Other	*P* value	−log⁡10(*P* value)
ASP	3.773832	4.229902	4.34*E* − 10	9.362655
Betweenness	0.002235	0.000337	2.59*E* − 25	24.58708
Closeness	0.270647	0.240556	3.86*E* − 15	14.4135
Clustering coefficient	0.061171	0.10591	0.088168	1.054689
Degree	30.52308	7.82407	8.74*E* − 36	35.05851
Eccentricity	9.323077	9.803829	9.55*E* − 08	7.019855
Radiality	0.801869	0.769293	4.34*E* − 10	9.362655
Stress	2551317	349608.7	1.47*E* − 29	28.83402
Topological coefficient	0.103758	0.201088	3.1*E* − 05	4.508613

**Table 5:** Diseases related to LWDH genes in OMIM.

Gene symbol	Location	MIM number	Disorders
ALB	4q13.3	103600	Analbuminemia; dysalbuminemic hyperthyroxinemia
APOA5	11q23.3	606368	Hypertriglyceridemia
BAX	19q13.33	600040	Colorectal cancer
BCL2	18q21.33	151430	Leukemia/lymphoma
KDR	4q12	191306	Hemangioma, capillary infantile
MET	7q31.2	164860	Renal cell carcinoma
PGR	11q22.1	607311	Progesterone resistance
PHB	17q21.33	176705	Breast cancer
